# Inflammatory Human Umbilical Cord-Derived Mesenchymal Stem Cells Promote Stem Cell-Like Characteristics of Cancer Cells in an IL-1*β*-Dependent Manner

**DOI:** 10.1155/2018/7096707

**Published:** 2018-02-18

**Authors:** Xiaohe Luo, Shan Huang, Ningning He, Chen Liu, Yanan Chen, Yanhua Liu, Xue Mi, Na Li, Peiqing Sun, Zongjin Li, Rong Xiang, Weijun Su

**Affiliations:** ^1^School of Medicine, Nankai University, Tianjin 300071, China; ^2^Department of Cancer Biology and Comprehensive Cancer Center, Wake Forest School of Medicine, Winston-Salem, NC 27157, USA; ^3^Institute of Radiation Medicine, Academy of Medical Science and Peking Union Medical College, Tianjin 300192, China; ^4^The First Affiliated Hospital of Chongqing Medical University, Chongqing 400016, China

## Abstract

To ensure the safety of clinical applications of MSCs, thorough understanding of their impacts on tumor initiation and progression is essential. Here, to further explore the complex dialog between MSCs and tumor cells, umbilical cord-derived mesenchymal stem cells (UC-MSCs) were employed to be cocultured with either breast or ovarian cancer cells. Though having no obvious influence on proliferation or apoptosis, UC-MSCs exerted intense stem cell-like properties promoting effects on both cancer models. Cocultured cancer cells showed enriched side population, enhanced sphere formation ability, and upregulated pluripotency-associated stem cell markers. Human cytokine array and real-time PCR revealed a panel of MSC-derived prostemness cytokines CCL2, CXCL1, IL-8, and IL-6 which were induced upon coculturing. We further revealed IL-1*β*, a well-characterized proinflammatory cytokine, to be the inducer of these prostemness cytokines, which was generated from inflammatory UC-MSCs in an autocrine manner. Additionally, with introduction of IL-1RA (an IL-1 receptor antagonist) into the coculturing system, the stem cell-like characteristics promoting effects of inflammatory UC-MSCs were partially blocked. Taken together, these findings suggest that transduced inflammatory MSCs work as a major source of IL-1*β* in tumor microenvironment and initiate the formation of prostemness niche via regulating their secretome in an IL-1*β*-dependent manner.

## 1. Introduction

Mesenchymal stem cells (MSCs) are a population of pluripotent stem cells which can be isolated from various organs and tissues, for example, bone marrow, adipose tissue, and umbilical cord [1]. The potential applications of MSCs in regenerative medicine and tumor targeting therapy have drawn much attention in recent years, by virtue of their capacity of differentiation and tumor-homing [2–4]. However, MSC-based therapy may be a double-edged sword, since a growing body of evidence implies that MSCs participate in both initiation and progression of tumors [5, 6]. The interactions between MSCs and tumor cells are complicated, and the mechanisms involved are multifaceted. Stemness is determinant for tumor initiation; thus the impacts of MSCs on stem cell-like properties of tumor cells need to be thoroughly understood before their clinical utilizations.

Heterogeneity exists among carcinoma cells within tumor, as a minority population of carcinoma cells, the so-called “cancer stem cells (CSCs),” possesses self-renewal and tumor initiating capacity which is deficient in the bulk [7]. Both extracellular matrix (ECM) and stromal cells in tumor microenvironment (TME) participate in the generation of a CSC niche. ECM works as the shelter of CSCs and the warehouse of protumorigenic elements [8]. Stromal cells, for example, macrophages, immune cells, and MSCs, are recruited to tumor sites and react to signaling from tumor cells by transforming into “cancer-favored” and reciprocally fuel CSCs [9–11].

The impacts of stromal cells on CSCs do not take place in void, but rather via either direct contacts or soluble factors. Inflammatory responses have long been found to be associated with various types of cancer and play decisive roles in cancer development [12]. IL-1*β*, a well-documented proinflammatory cytokine, is suggested to be highly relevant with the inflammatory setup in several tumor types and is correlated with higher ratio of relapse and disease progression [13, 14]. Some tumors, including advanced melanoma, spontaneously release IL-1*β* [15]. Tumor infiltrating macrophages and neutrophils are also found to be dominant sources of IL-1*β* in TME, and treatments with IL-1*β* receptor antagonist lead to the delay of tumor formation [16].

To further delineate the complex dialog between MSCs and tumor cells, MSCs were isolated from human umbilical cord (UC-MSCs), and coculturing model was employed to study their impacts on cancer cells. Our study reveals that secretome of cocultured UC-MSCs apparently enhances stem cell-like characteristics of cancer cells, which is dependent on IL-1*β* secretion of inflammatory UC-MSCs.

## 2. Materials and Methods

### 2.1. Cell Culture

MDA-MB-231 human breast cancer cells and IGROV1 human ovarian cancer cells were cultured with DMEM (high glucose) medium (Corning, Lowell, MA) supplemented with 10% fetal bovine serum (FBS) (Corning, Lowell, MA) and 1% penicillin streptomycin solution (Gibco, Rockville, MD). Medium for MDA-MB-231 cells was also supplemented with 1% MEM nonessential amino acid solution (NEAA; Gibco). UC-MSCs were isolated as described before [17, 18] and cultured with DMEM/F12 medium (Gibco) containing 10% FBS (Corning), 1% penicillin streptomycin solution (Gibco), and 10 ng/ml human recombinant epidermal growth factor (EGF; Gibco). All cell lines were maintained at 37°C in a 5% CO2 incubator. To be trackable in direct coculturing model, MDA-MB-231 cells were transduced with lentiviral vector carrying green fluorescence protein (GFP) and selected with blasticidin.

### 2.2. Collection of Conditioned Medium

MDA-MB-231 cells, IGROV1 cells, or UC-MSCs were cultured to 70–80% confluency in T75 flasks, and the medium was replaced with 10 ml fresh basic medium per flask, respectively. 24 hours later, conditioned medium was collected, aliquoted, and stored in −80°C until use.

### 2.3. Coculturing of Cancer Cells and UC-MSCs

For indirect coculturing model, on the first day, UC-MSCs were treated with 10 *μ*g/ml mitomycin C diluted in culture medium for 1.5 hours to suppress proliferation. Treated UC-MSCs were seeded onto the 0.4 *μ*m Transwell upper chamber at a density of 3 × 10^5^ cells/well for 6-well plate. Cancer cells were seeded at a density of 3 × 10^5^ cells/well for 6-well plate. On the second day, after cells were attached, Transwell chambers with attached UC-MSCs were placed onto the plates with attached cancer cells. Experiments were performed after 3-day coculturing.

### 2.4. Immunofluorescence Staining

For immunofluorescence staining, specific antibodies against CD29 (Abcam, Cambridge, MA), CD44 (Abcam), CD90 (Abcam), and CD105 (Abcam) followed by Alexa Fluor 488/594 labeled-secondary antibodies (Invitrogen, Carlsbad, CA) were used for detection. Cells were counterstained with DAPI, mounted with antifade mounting medium, and photographs were taken under fluorescence microscope.

### 2.5. Flow Cytometry Analysis

For detection of mesenchymal stem cell surface markers, specific antibodies against CD44 (Abcam), CD90 (Abcam), and CD105 (Abcam) followed by FITC labeled-secondary antibody were used. After the staining process, cells were resuspended with PBS supplemented with 2% FBS and analyzed with FACS.

For side population analysis, MDA-MB-231 and IGROV1 cells in 6-well plates were rinsed with PBS, and 5 *μ*g/ml Hoechst 33342 staining buffer was added onto the cells. The plates were placed into 37°C incubator for staining. 1 h later, the Hoechst 33342 staining buffer was removed, and the cells were rinsed with PBS and digested with trypsin. Cells were resuspended with PBS supplemented with 2% FBS and analyzed with FACS. For MDA-MB-231 cells, 10 *μ*g/ml reserpine was used as blocker. For IGROV1 cells, 10 *μ*g/ml verapamil was used as blocker.

### 2.6. Differentiation of UC-MSCs

Differentiation of UC-MSCs into adipocytes was performed using Mesenchymal Stem Cell Adipogenic Differentiation Medium (Cyagen, HUXUC-90031) according to the manufacturer's instruction. The Oil Red O staining was performed 17 days later. Differentiation of UC-MSCs into chondrocytes was performed using Mesenchymal Stem Cell Chondrogenic Differentiation Medium (Cyagen, HUXUC-9004) according to the manufacturer's instruction. The tissue samples were formalin-fixed and paraffin-embedded on day 18 and further underwent Alcian Blue staining. Differentiation of UC-MSCs into osteoblasts was performed using Mesenchymal Stem Cell Osteoblastic Differentiation Medium (Cyagen, HUXUC-90021) according to the manufacturer's instruction. The Alizarin Red staining was performed 17 days later.

### 2.7. Proliferation Assay

Cancer cells were seeded into 96-well plate at 5 × 10^3^ cells/well on the first day and cultured with a mixture of UC-MSC conditioned medium and cancer cell fresh medium at a ratio of 1 : 1 supplemented with 10% FBS and 1% penicillin streptomycin solution. CCK-8 assay kit bought from Dojindo Molecular Technologies (Kumamoto, Japan) was used for assessment. Before each measurement, the culture medium was replaced with 100 *μ*l fresh medium per well. 10 *μ*l CCK-8 working solution was added to each well, and cells were incubated for another 2 hours at 37°C in a 5% CO2 incubator. 2 hours later, the absorbance at 450 nm was measured using GloMax-Multi Detection System (Promega, Madison, WI) and analyzed.

For detection of Ki67 expression, MDA-MB-231 or IGROV1 cells indirectly cocultured with UC-MSCs were harvested at 48 h or 72 h. Specific antibody against Ki67 (Abcam), followed by Alexa Fluor 488 labeled-secondary antibodies (Invitrogen), was used for detection. Cells were counterstained with DAPI, mounted with antifade mounting medium, and photographs were taken under fluorescence microscope.

### 2.8. Apoptosis Assay

After being cocultured with UC-MSCs, cancer cells were prepared using Annexin V-fluorescein isothiocyanate (FITC) apoptosis detection kit (Keygene, Nanjing, China) according to the manufacturer's instructions, and stained cells were analyzed by FACS.

### 2.9. Sphere Formation Assay

After being indirectly cocultured with UC-MSCs, 1 × 10^4^ MDA-MB-231 cells were resuspended with sphere formation medium (DMEM basic medium supplemented with 20 ng/ml hEGF, 20 ng/ml bFGF, and 1 × B27 supplement) and seeded into Ultralow Attachment 6-well plate (Corning). 5 days later, photographs were taken under microscope, and spheres with 50–250 *μ*m diameter were counted and analyzed.

### 2.10. Cytokine Array

MDA-MB-231 cells and UC-MSCs were indirectly cocultured in 6-well plates as mentioned above. Medium was replaced with 5 ml fresh basic medium (a mixture of 4 ml DMEM and 1 ml DMEM/F12 medium with 1% penicillin streptomycin solution per well) after three-day coculturing. For the control group, the same procedure was performed only without seeding UC-MSCs onto the upper Transwell chamber. The supernatant was collected 24 hours later and underwent cytokine assay immediately.

Proteome Profiler™ Human Cytokine Array Kit (ARY005B, R&D Systems, Minneapolis, MN) was used for cytokine array. 1 ml supernatant per sample was used for cytokine array. The array was performed according to the manufacturer's instruction, and the membranes were exposed and analyzed.

### 2.11. Treatment with Cytokines or Antagonists

Treatment with cytokines in MDA-MB-231 or UC-MSCs was performed with recombinant human IL-1*β* (10139-HNAE, Sino Biological Inc., Beijing, China) at 1 ng/ml, recombinant human CCL2 (10134-H08Y, Sino Biological Inc.) at 100 ng/ml, and recombinant human CXCL1 (10877-HNCE, Sino Biological Inc.) at 100 ng/ml. For the treatment with antagonist, recombinant human IL-1RA (10123-HNAE, Sino Biological Inc.) was added to 231-MSC coculturing system at 10 *μ*g/ml. Total RNA or proteins were collected 24 h after the treatments, and further analysis was performed.

### 2.12. Western Blots

Cells lysates were prepared with RIPA buffer, mixed with loading buffer, and boiled at 100°C for 10 min for denaturation. Cleared cell lysates were subjected to SDS-PAGE gel and transferred to PVDF membrane. Proteins were examined with anti-Sox2 (sc-20088, Santa Cruz, Dallas, Texas), anti-Oct4 (ab19857, Abcam), anti-KLF4 (4038, Cell Signaling Technology, Danvers, MA), and anti-*β*-actin (sc-130300, Santa Cruz) antibodies diluted in 5% BSA buffer. Reactions were detected using Immobilon Western Chemiluminescent HRP Substrate (Millipore, Billerica, MA).

### 2.13. Real-Time PCR

Total RNA was extracted with Trizol™ reagent (Invitrogen) and reverse transcribed with PrimeScript RT reagent (TaKaRa, Shiga, Japan). Real-time PCR was performed using SYBR Premix Ex Taq (TaKaRa) on Lightcycler 96 Real-Time PCR System (Roche, Indianapolis, IN) with the following primers: human CCL2, 5′-CAGCCAGATGCAATCAATGCC-3′ (forward) and 5′-TTTGCTTGTCCAGGTGGTCC-3′ (reverse); human CXCL1, 5′-GCAGGGAATTCACCCCAAGA-3′ (forward) and 5′-TGGATTTGTCACTGTTCAGCA-3′ (reverse); human IL-8, 5′-TGTGAAGGTGCAGTTTTGCCA-3′ (forward) and 5′-CAACCCTCTGCACCCAGTTT-3′ (reverse); human IL-6, 5′-CAATGAGGAGACTTGCCTGGT-3′ (forward) and 5′-ATTTGTGGTTGGGTCAGGGG-3′ (reverse); human IL-1*β*, 5′-ATGATGGCTTATTACAGTGGCA-3′ (forward) and 5′-CATGGCCACAACAACTGACG-3′ (reverse); human GAPDH, 5′-CTCTGATTTGGTCGTATTGGG-3′ (forward) and 5′-TGGAAGATGGTGATGGGATT-3′ (reverse).

### 2.14. Transwell Migration Assay

MDA-MB-231 cells were treated with CCL2 or CXCL1 as indicated. 1 × 10^5^ treated MDA-MB-231 cells or control cells were resuspended in 200 *μ*l basic medium and seeded upon Millicell Hanging Cell Culture Inserts (Millipore) with attractants in the lower chamber. After incubation for 8 hours, the inserts were taken out, and the cells were removed from the upper chamber with a cotton swab. Cells which have migrated through the pores and attached to the reverse side of the membrane were fixed with 4% formaldehyde in PBS, followed by staining with 0.5% crystal violet for 20 min. Cells were counted under the microscope at 200x and statistically analyzed.

### 2.15. ELISA Assay

Supernatant of 231-MSC coculture was collected as mentioned above, and 20 ul supernatant per sample was used for ELISA assay. ELISA assay of IL-1*β* was performed using human IL-1*β* ELISA kit (EK101B2, Lianke Bio Inc., Hangzhou, China) following the manufacturer's instruction. OD value at 450 nm was detected with GloMax-Multi Detection System (Promega), and absolute IL-1*β* concentration was calculated according to the standard curve.

### 2.16. Statistical Analysis

Statistics were calculated using SigmaStat for Windows Version 3.5 (Systat, San Jose, CA, USA). For comparison between two groups, two-tailed Student's *t*-test was used. Differences were considered significant at values of *p* < 0.05.

## 3. Results

### 3.1. Characteristics of Human Umbilical Cord-Derived Mesenchymal Stem Cells (UC-MSCs)

**Figure 1:**
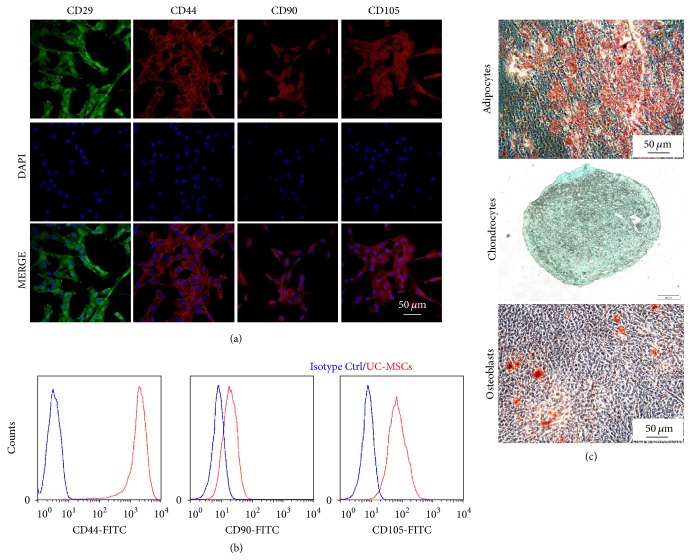
*Characteristics of UC-MSCs.* (a) Immunofluorescent staining of CD29, CD44, CD90, and CD105 in human umbilical cord-derived MSCs (UC-MSCs). (b) Flow cytometry analysis of CD44, CD90, and CD105 expression in UC-MSCs. (c) Differentiation of UC-MSCs into 3 distinct lineages, namely, adipocytes, chondrocytes, and osteoblasts.

It is well known that mesenchymal stem cells (MSCs) can be isolated from various sources, for example, bone marrow and adipose tissue. In our study, MSCs were isolated from human umbilical cord following the protocol described before [17, 18]. The isolated cells were adherent to tissue culture plastic, had fibroblast-like morphology, and proliferated rapidly (data not shown). To further verify the MSC characteristics, immunofluorescence staining of CD29, CD44, CD90, and CD105 was performed in these cells. As shown in Figure 1(a), all isolated umbilical cord-derived mesenchymal stem cells (UC-MSCs) showed the expression of these MSC markers, which indicates MSC properties of the isolated cells. This was further verified by FACS analysis of these markers (Figure 1(b)). And the isolated UC-MSCs also have differentiation potential into 3 distinct lineages, namely, adipocytes, chondrocytes, and osteoblasts (Figure 1(c)).

### 3.2. UC-MSCs Have No Impact on the Proliferation or Apoptosis of Cancer Cells

**Figure 2:**
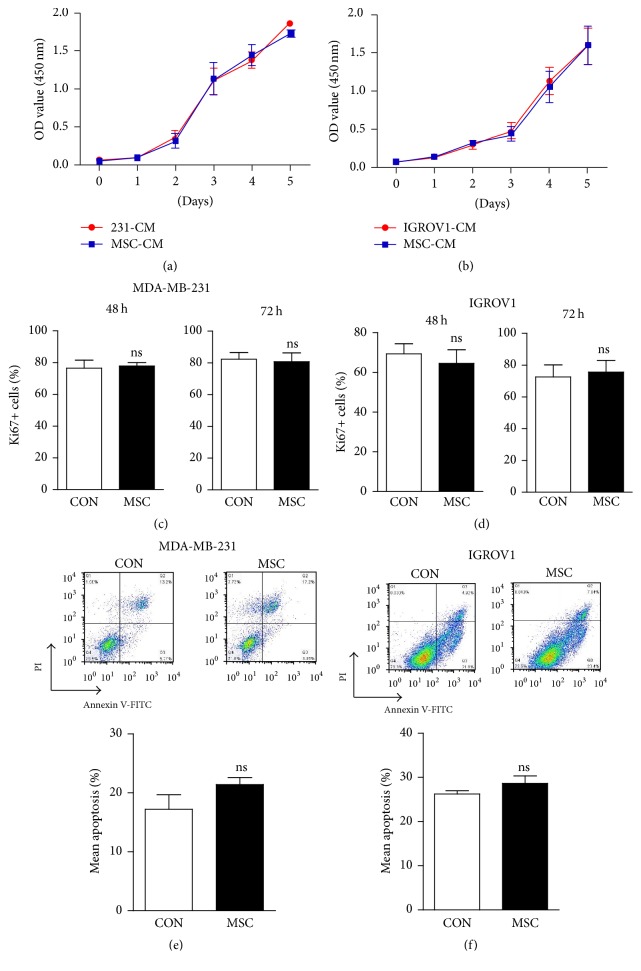
*Effects of UC-MSCs on proliferation and apoptosis of cancer cells.* (a-b) UC-MSC conditioned medium (MSC-CM) 1 : 1 mixed with fresh basic medium was supplemented with 10% FBS and used for culturing. CCK8 assay was performed in MDA-MB-231 (a) or IGROV1 cells (b) at the indicated time points, and OD value was measured at 450 nm. (c-d) MDA-MB-231 (c) or IGROV1 cells (d) were indirectly cocultured with UC-MSCs and harvested at the indicated time points, and the percentage of Ki67 positive cells was shown here. *n* = 3; ns stands for nonsignificance. (e-f) Annexin V-PI staining was performed in MDA-MB-231 (e) or IGROV1 cells (f) cocultured with UC-MSCs, and apoptotic cells were analyzed by FACS. Representative dot plots and statistical results were shown here. *n* = 3; ns stands for nonsignificance.

Tumor promoting effects of MSCs from various sources have been reported by a series of literatures, either by proproliferation and promoting epithelial-mesenchymal transition (EMT) or via regulating TME [19–21]. However, in our study, proliferation rate of breast or ovarian cancer cells cultured with conditioned medium of UC-MSCs has no significant difference with control cells (Figures 2(a) and 2(b)). To further investigate the effects of MSCs on proliferation of cancer cells, we also performed indirect coculturing model in both MDA-MB-231 and IGROV1 cells. As shown in Figures 2(c) and 2(d), upon coculturing with UC-MSCs, Ki67 positive rates in neither MDA-MB-231 nor IGROV1 cells showed significant changes. And coculturing with UC-MSCs had no obvious impacts on apoptosis in breast or ovarian cancer cells (Figures 2(e) and 2(f)).

### 3.3. UC-MSCs Promote Stem Cell-Like Characteristics in Cancer Cells

Though UC-MSCs have no apparent impact on either proliferation or apoptosis of cancer cells, we found that UC-MSCs influenced the growth pattern of cancer cells. Upon direct coculturing with UC-MSCs, MDA-MB-231 cells formed large mammosphere-like structures suspending in culture medium (data not shown). This phenomenon implies that UC-MSCs may possibly promote stem cell-like properties of cancer cells.

**Figure 3:**
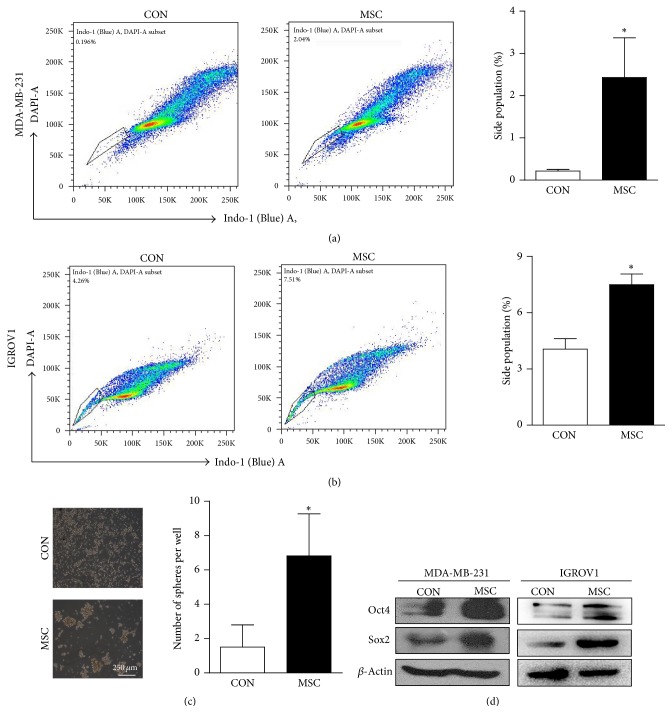
*UC-MSCs enhance stem cell-like characteristics in cancer cells.* (a-b) Hoechst staining was performed in MDA-MB-231 cells (a) and IGROV1 cells (b) indirectly cocultured with UC-MSCs, and statistical results of side population (SP) by FACS were shown here. *n* = 3 for MDA-MB-231 cells, and *n* = 2 for IGROV1 cells, ^*∗*^*p* < 0.05. (c) After being indirectly cocultured with UC-MSCs, MDA-MB-231 cells were harvested and seeded into low attachment plates for sphere formation assay. Representative images and statistical results were shown here. *n* = 4, ^*∗*^*p* < 0.05. (d) Expression levels of pluripotency markers Sox2 and Oct4 in MDA-MB-231 and IGROV1 cells indirectly cocultured with UC-MSCs by Western blots.

To investigate whether CSC ratio can be increased by UC-MSCs, Hoechst staining and sphere formation assay were performed in cancer cells indirectly cocultured with UC-MSCs. Indirect coculturing helps avoid direct contacts and possible mechanical influences from UC-MSCs. We found that the percentage of side population (SP), which represents the subpopulation of cancer cells possessing stem cell-like properties, dramatically increased from 0.1% to 2% in MDA-MB-231 cells after coculturing (Figure 3(a)), and similar tendency was seen in IGROV1 ovarian cancer cells (Figure 3(b)). In sphere formation assay, coculturing with UC-MSCs increased the sphere formation ability of MDA-MB-231 cells after being indirectly cocultured with UC-MSCs (Figure 3(c)). Furthermore, Western blots showed that the overall expression levels of pluripotency markers Sox2 and Oct4 in both MDA-MB-231 and IGROV1 cells were greatly upregulated after indirect coculturing with UC-MSCs (Figure 3(d)). All these phenomena implied the promotion of stem cell-like properties in cancer cells by UC-MSCs.

### 3.4. UC-MSCs Derived Cytokines Upregulate Expression of Pluripotency Markers and Promote Migration of Breast Cancer Cells

**Figure 4:**
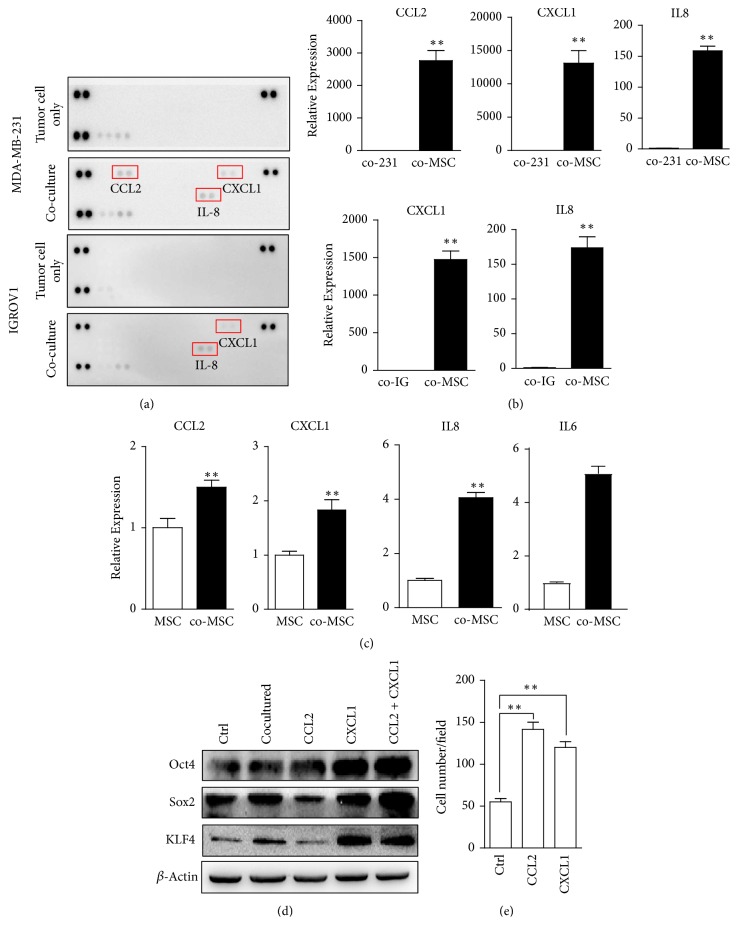
*Cytokines derived from cocultured UC-MSCs enhance stem cell-like characteristics in cancer cells.* (a) Detection of increased cytokines in 231-MSC coculture compared to MDA-MB-231 cell culture and IGROV1-MSC coculture compared to IGROV1 cell culture by human cytokine array. (b) Relative expression levels of CCL2, CXCL1, and IL-8 in MDA-MB-231 cells and UC-MSCs in 231-MSC coculture and relative expression of CXCL1 and IL-8 in IGROV1 cells and UC-MSCs in IGROV1-MSC coculture. ^*∗∗*^*p* < 0.01. (c) Relative expression levels of CCL2, CXCL1, IL-8, and IL-6 in UC-MSCs cocultured with MDA-MB-231 cells compared to UC-MSCs cultured alone. ^*∗∗*^*p* < 0.01. (d) Effects of CCL2 and CXCL1 on pluripotency markers Sox2, Oct4, and KLF4 expression in MDA-MB-231 cells. (e) Effects of CCL2 and CXCL1 on migration ability of MDA-MB-231 cells by Transwell migration assay. *n* = 3, ^*∗∗*^*p* < 0.01.

To investigate which soluble factors play roles in the promotion of stem cell-like characteristics, human cytokine array was performed with the supernatant of indirect coculturing system. Among 36 cytokine candidates, CCL2, CXCL1, and IL-8 levels were greatly enhanced in 231-MSC coculture, and CXCL1 and IL-8 levels were enhanced in IGROV1-MSC coculture (Figure 4(a)). And UC-MSCs instead of cancer cells under coculturing are the dominant sources of these cytokines, as indicated in Figure 4(b). Since the enrichment of side population in MDA-MB-231 cells upon indirectly coculturing with MSCs was more obvious when compared to IGROV1 and the increase of cytokines was more significant, we mainly focused on MDA-MB-231 cells in the following studies.

Comparing UC-MSCs under coculturing and those cultured alone, we found that coculturing upregulated CCL2, CXCL1, and IL-8 expression, as well as another well-known prostemness cytokine IL-6 in UC-MSCs (Figure 4(c)). This implies that secretome of UC-MSCs was regulated by cocultured cancer cells.

Since IL-8 and IL-6 are already well-known prostemness cytokines [22, 23], we further assessed the effects of CCL2 and CXCL1 on stem cell-like properties. Treatment with CCL2 or CXCL1 upregulated expression levels of pluripotency markers Sox2, Oct4, and KLF4 in MDA-MB-231 cells and had synergic effects (Figure 4(d)). Furthermore, treatment with CCL2 or CXCL1 enhanced the migration ability of MDA-MB-231 cells in Transwell assay (Figure 4(e)). Together with IL-6 and IL-8, the panel of cytokines derived from inflammatory UC-MSCs built up a prostemness niche collectively.

### 3.5. Inflammatory UC-MSCs Secrete IL-1*β* in an Autocrine Manner and Generate a Prostemness Niche via Regulating Secretome

**Figure 5:**
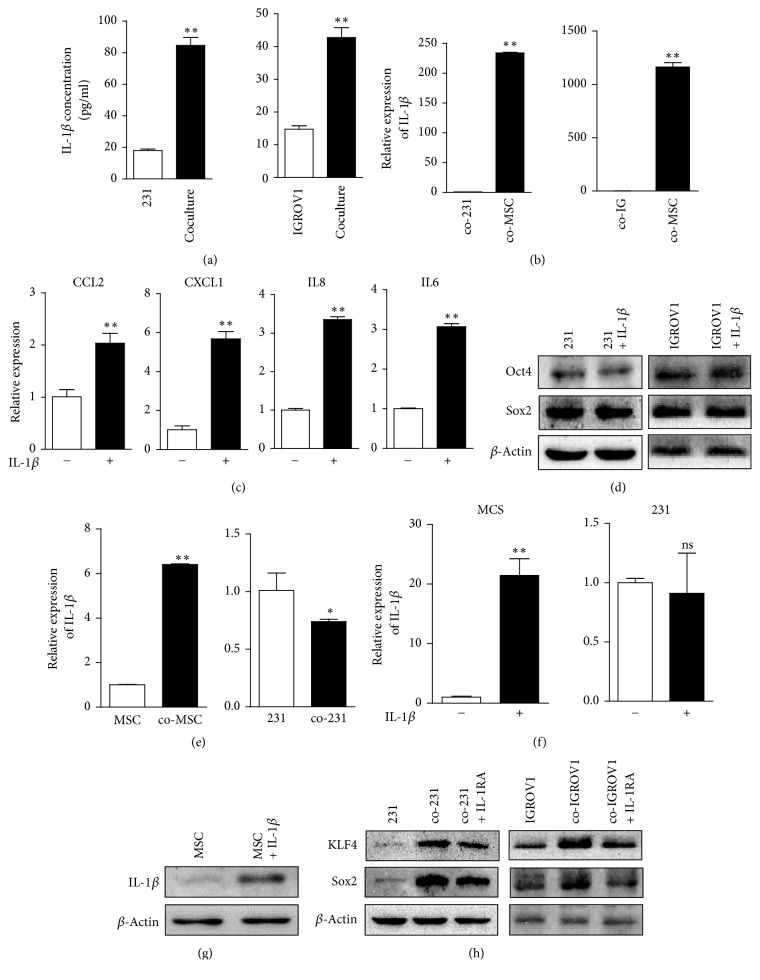
*Inflammatory UC-MSCs secrete IL-1β in an autocrine manner and generate a prostemness niche via upregulating CCL2, CXCL1, IL-8, and IL-6.* (a) Comparison of IL-1*β* concentration between MDA-MB-231 cell culture and 231-MSC coculture, and between IGROV1 cell culture and IGROV1-MSC coculture by ELISA assay. ^*∗∗*^*p* < 0.01. (b) Relative expression level of IL-1*β* in MDA-MB-231 cells and UC-MSCs in 231-MSC coculture and in IGROV1 cells and UC-MSCs in IGROV1-MSC coculture. ^*∗∗*^*p* < 0.01. (c) Expression levels of CCL2, CXCL1, IL-8, and IL-6 in UC-MSCs upon IL-1*β* treatment. ^*∗∗*^*p* < 0.01. (d) Expression levels of pluripotency markers Sox2 and Oct4 in MDA-MB-231 and IGROV1 cells upon IL-1*β* treatment. (e) Relative expression level of IL-1*β* in UC-MSCs and MDA-MB-231 cells under coculturing compared to cells cultured alone. ^*∗∗*^*p* < 0.01; ^*∗*^*p* < 0.05. (f) mRNA level of IL-1*β* upon IL-1*β* treatment in UC-MSCs and MDA-MB-231 cells. ^*∗∗*^*p* < 0.01; ns stands for nonsignificance. (g) Protein level of IL-1*β* upon IL-1*β* treatment in UC-MSCs. (h) Effects of IL-1RA on the expressions of Sox2 and KLF4 in MDA-MB-231 and IGROV1 cells under coculturing. ns, nonsignificance.

The uniform increase of prostemness cytokines in UC-MSCs upon coculturing led us to consider whether there were upstream cytokines playing roles in the process. IL-1*β*, one dominant product of inflammasome, is a well-characterized proinflammatory cytokine and has been reported to upregulate CXCL1, IL-8, and IL-6, respectively [16, 24, 25]. Via ELISA assay, higher level of IL-1*β* was detected in the 231-MSC coculture, as well as in IGROV1-MSC coculture (Figure 5(a)), which was derived from UC-MSCs rather than cancer cells (Figure 5(b)). No detection of IL-1*β* increase in human cytokine array may be due to its relatively lower level in the supernatant.

Upon recombinant human IL-1*β* treatment, expression levels of prostemness cytokines CCL2, CXCL1, IL-8, and IL-6 were upregulated in UC-MSCs (Figure 5(c)), whereas IL-1*β* itself could not affect pluripotency markers expression in either MDA-MB-231 or IGROV1 cells (Figure 5(d)). It indicates that IL-1*β* derived from UC-MSCs promotes stem cell-like characteristics via regulating secretome of UC-MSCs, instead of playing a direct role in cancer cells.

In addition, coculturing induced higher expression level of IL-1*β* in UC-MSCs, but not in MDA-MB-231 (Figure 5(e)). Upon treatment with IL-1*β*, both mRNA and protein level of IL-1*β* are increased in UC-MSCs, with no significant impact in MDA-MB-231 cells (Figures 5(f) and 5(g)). This implies a positive feedback loop of IL-1*β* in UC-MSCs.

### 3.6. IL-1 Receptor Antagonist Blocks the Prostemness Effects of UC-MSCs

To further demonstrate the indirect prostemness function of IL-1*β* derived from UC-MSCs, IL-1 receptor antagonist (IL-1RA) was introduced into the coculturing system. IL-1RA, as a member of the interleukin 1 cytokine family, binds nonproductively to IL-1R, thus inhibiting the function of IL-1*β* [26]. IL-1RA added to the coculturing system partially blocked the upregulation of tumor-associated pluripotency factors Sox2 and KLF4 in both breast cancer cells and ovarian cancer cells (Figure 5(h)), which verified the role of IL-1*β* in the promotion of stem cell-like properties.

**Figure 6:**
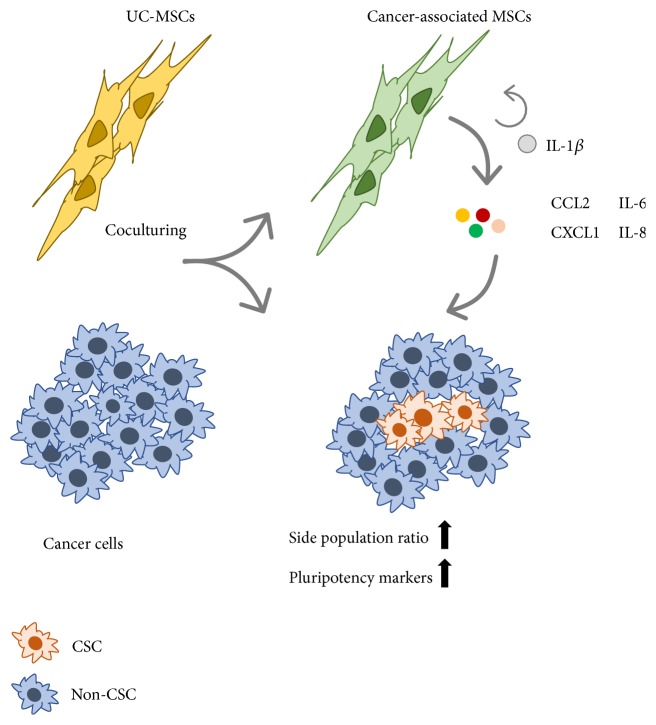
*A putative model for prostemness characteristics of inflammatory UC-MSCs cocultured with cancer cells.* Upon coculturing with cancer cells, UC-MSCs possess inflammatory phenotype with enhanced IL-1*β* level. IL-1*β* derived from inflammatory UC-MSCs creates a prostemness niche by upregulating CCL2, CXCL1, IL-8, and IL-6 secretion in UC-MSCs in an autocrine manner, which reciprocally function on cancer cells, enhance side population (SP) ratio, and upregulate pluripotency markers.

Taken together, upon coculturing with cancer cells, UC-MSCs obtain inflammatory phenotype with enhanced secretion of IL-1*β*. MSC-derived IL-1*β* establishes a prostemness niche by inducing the secretion of CCL2, CXCL1, IL-8, and IL-6 from UC-MSCs. These downstream cytokines reciprocally work on cancer cells, increase side population ratio, and upregulate expression level of stemness markers. And the promotion of stem cell-like characteristics of UC-MSCs on cancer cells can be partially blocked by IL-1R antagonist IL-1RA (Figure 6).

## 4. Discussion

UC-MSCs are considered as attractive tools for tumor targeting therapy and regenerative medicine in recent years, partially because of their noninvasive collection procedure and expanding ability [27–29]. However, their potential tumorigenic properties raise safety concerns, and the detailed effects of UC-MSCs in tumor progression merit further investigations. It has been well accepted that stromal cells play indispensable roles in tumor development and progression. MSCs from diverse resources, for example, bone marrow and adipose tissue, can be recruited into tumor sites and interact with parenchymal cells via both direct contact and soluble factors [30, 31]. Our work shows that IL-1*β* derived from inflammatory UC-MSCs induces the production of downstream cytokines CCL2, CXCL1, IL-6, and IL-8 in an autocrine manner, which promotes stem cell-like characteristics of cocultured cancer cells.

Though having no influence on proliferation or apoptosis, cocultured UC-MSCs greatly enhance SP ratio in both breast and ovarian cancer cells, as well as upregulating the well-characterized tumor-associated pluripotency factors Sox2 and Oct4. Likewise, several other studies present evidences of the involvement of MSCs in CSC maintenance. For instance, MSCs were shown to cause aberrant microRNA profile in breast cancer cells, which promotes CSC propagation via repression of FOXP2 [32]. Different soluble factors and signaling pathway were suggested to be involved in the process. CXCL7- IL-6 loop and Hedgehog- (HH-) BMP4 loop are found to be dominant mediators in the maintenance of CSCs by MSCs under different conditions [33, 34]. The release of PGE2 and its induced cytokines from MSCs is also found to participate in the creation of cancer stem cell niche, the process of which is triggered by Il-1*β* in TME [24]. As a well-characterized proinflammatory cytokine, IL-1*β* is associated with more aggressive phenotype and higher tumor grade in breast cancer [35], and genetic variations of IL-1*β* and IL-1R1 may predict breast cancer risk and prognosis [36, 37]. Different from the observation that IL-1*β* is secreted dominantly from cancer cells in TME [24, 25], in our current study, inflammatory UC-MSCs are the major source of IL-1*β* in the coculturing system. Besides, upon treatment with IL-1*β*, expression level of IL-1*β* was greatly enhanced in UC-MSCs, but not in breast cancer cells, implying a positive feedback loop in MSCs. Whether the inflammasome activation is also involved in this process still needs further investigations. All these phenomena indicate that intervention of IL-1*β* expression in MSCs is a potential approach to avoid possible side effects in clinical utilization.

Besides Il-1*β* and its downstream cytokines, there are other mechanisms which may participate in the promotion of stem cell-like properties in tumor cells by MSCs. In the present study, breast cancer cells directly cocultured with UC-MSCs were found to form mammosphere-like constructs suspending in culture, the mechanism of which may involve both soluble factors and direct interactions. The possibility that fusion and entosis take place between stromal cells and tumor cells and form hybrids exhibiting CSC properties has been suggested [30, 38, 39]. Furthermore, direct communications between stromal cells and tumor cells via gap junctional intercellular communication (GJIC), nanotubes, and trogocytosis are also considered to contribute to the strength of stem cell-like characteristics [30, 39–41]. For examples, it has been reported that multidrug resistance protein can be extracted by cancer cells from stromal cells [41]. Whether similar mechanisms are involved in the interactions between tumor cells and UC-MSCs still needs further investigations.

In summary, our present study provides a new insight that inflammatory MSCs are a dominant source of IL-1*β* in TME and can trigger the formation of prostemness niche via regulating secretome in an IL-1*β*-dependent manner. This result implies that IL-1*β* plays a significant role in tumorigenic functions of UC-MSCs, which should be eradicated to ensure the safety of their clinical applications.

## Abbreviations

UC-MSCs: Umbilical cord-derived mesenchymal stem cells
CSCs: Cancer stem cells
ECM: Extracellular matrix
TME: Tumor microenvironment
GFP: Green fluorescent protein
EMT: Epithelial-mesenchymal transition
SP: Side population.

## Supplementary Materials

Supplementary File To investigate the effects of MSCs on proliferation of cancer cells, we performed immunofluorescent staining for Ki67 in cancer cells indirectly cocultured with UC-MSCs and found no significant influence on Ki67 positive percentage in either MDA-MB-231 or IGROV1 cells. Representative pictures were shown in Supplementary Data 1A-B, related to Figures  2(c)-2(d). For side population analysis, both MDA-MB-231 and IGROV1 cells were stained with 5 *μ*g/ml Hoechst 33342. In MDA-MB-231 and IGROV1 cells, 10 *μ*g/ml reserpine and 10 *μ*g/ml verapamil were used as blockers, respectively. The representative FACS dot plots for each cell type were shown in Supplementary Data 2A-B. The repeats of side population analysis in MDA-MB-231 cells cocultured with UC-MSCs were shown in Supplementary Data 3, and the repeats of side population analysis in IGROV1 cells cocultured with UC-MSCs were shown in Supplementary Data 4.
