# Supplementary material for: Inflammatory Human Umbilical Cord-Derived Mesenchymal Stem Cells Promote Stem Cell-Like Characteristics of Cancer Cells in an IL-1β-Dependent Manner
Source: Biomed Res Int. 2018 Feb 18;2018:7096707. doi: 10.1155/2018/7096707 (PMC5835289; doi:10.1155/2018/7096707)
Supplement: Supplementary File — To investigate the effects of MSCs on proliferation of cancer cells, we performed immunofluorescent staining for Ki67 in cancer cells indirectly cocultured with UC-MSCs and found no significant influence on Ki67 positive percentage in either MDA-MB-231 or IGROV1 cells. Representative pictures were shown in Supplementary Data 1A-B, related to Figures 2(c)-2(d). For side population analysis, both MDA-MB-231 and IGROV1 cells were stained with 5 μg/ml Hoechst 33342. In MDA-MB-231 and IGROV1 cells, 10 μg/ml reserpine and 10 μg/ml verapamil were used as blockers, respectively. The representative FACS dot plots for each cell type were shown in Supplementary Data 2A-B. The repeats of side population analysis in MDA-MB-231 cells cocultured with UC-MSCs were shown in Supplementary Data 3, and the repeats of side population analysis in IGROV1 cells cocultured with UC-MSCs were shown in Supplementary Data 4. [file 7096707.f1.pdf]

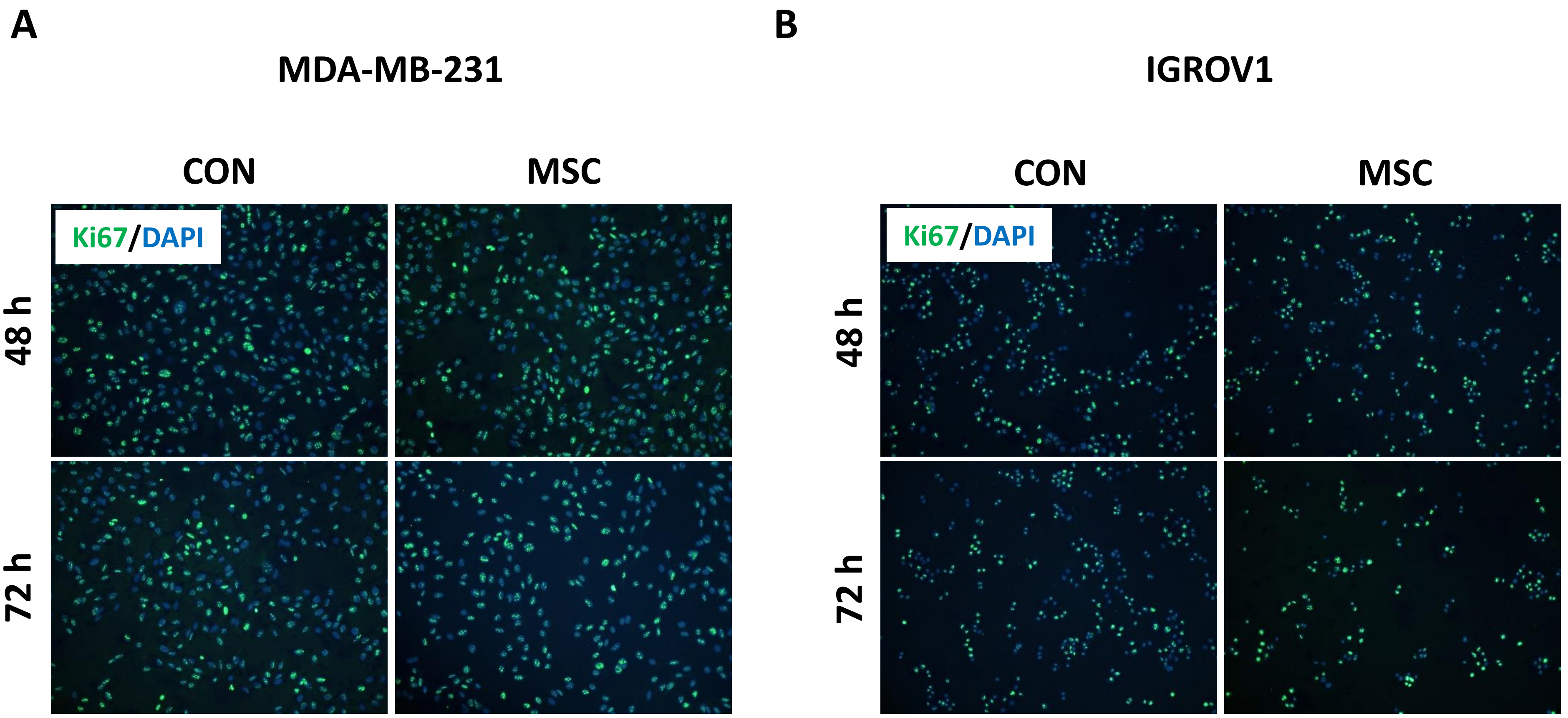

Supplementary Data 1

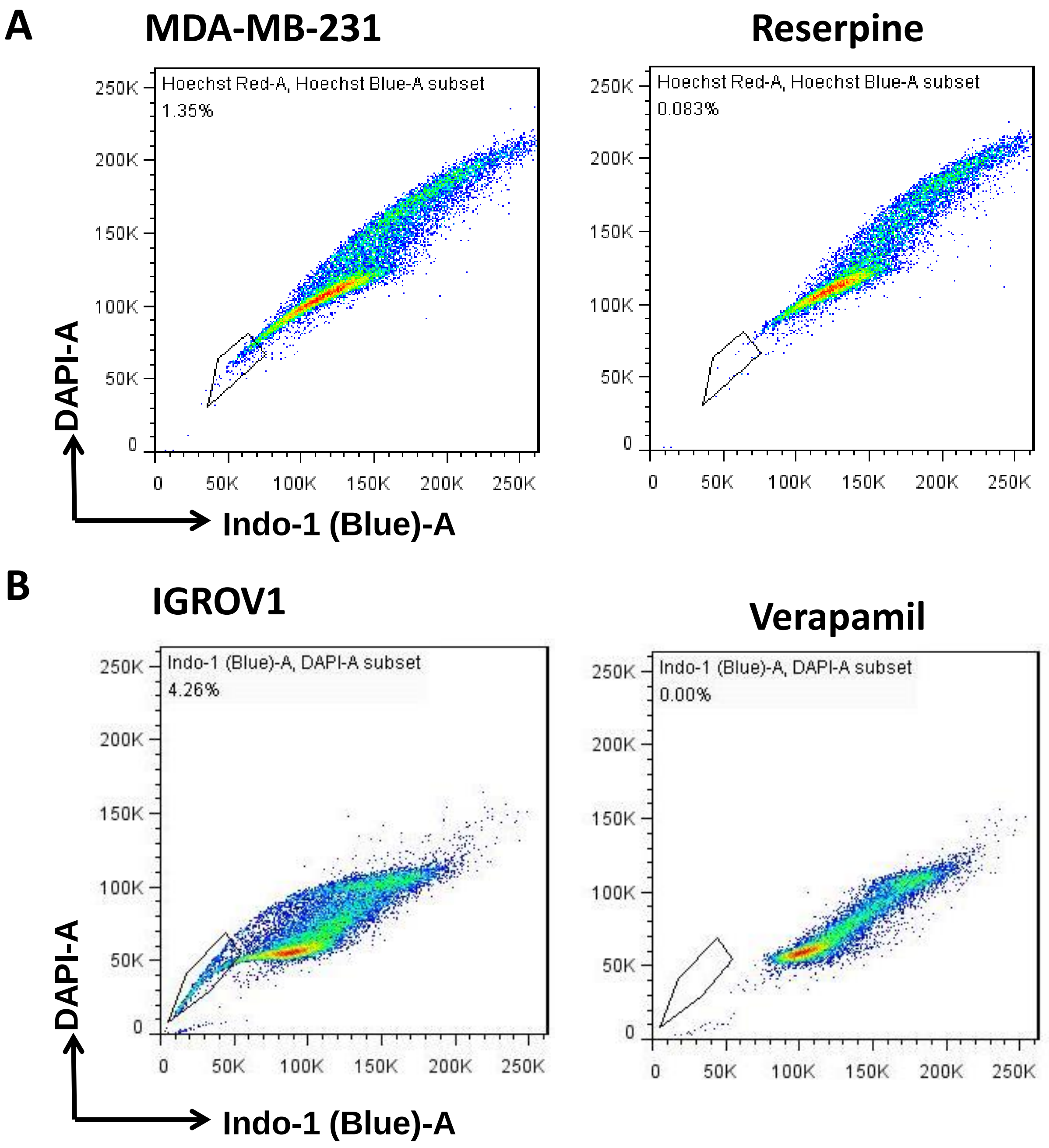

Supplementary Data 2

MDA-MB-231

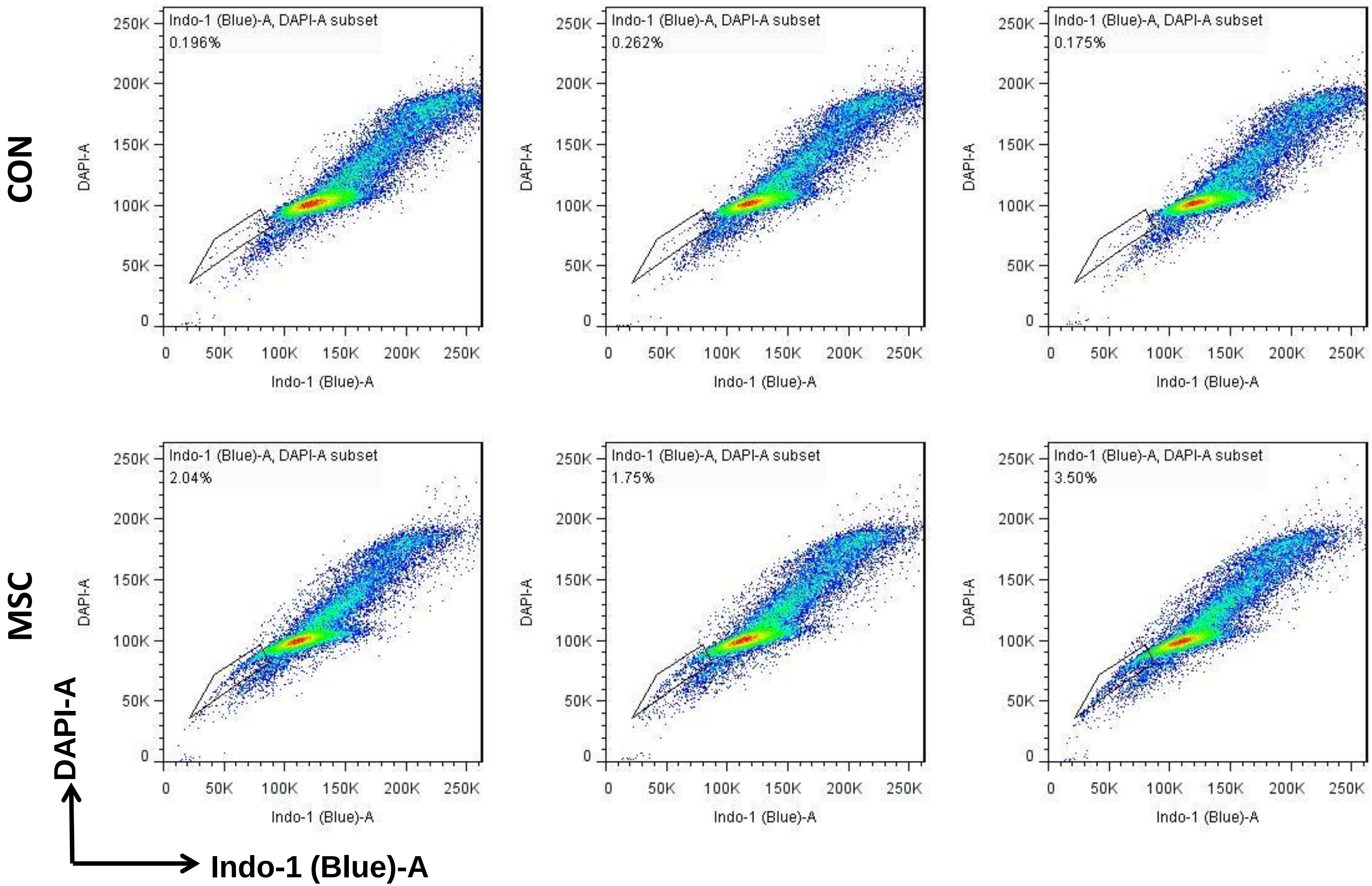

Supplementary Data 3

IGROV1

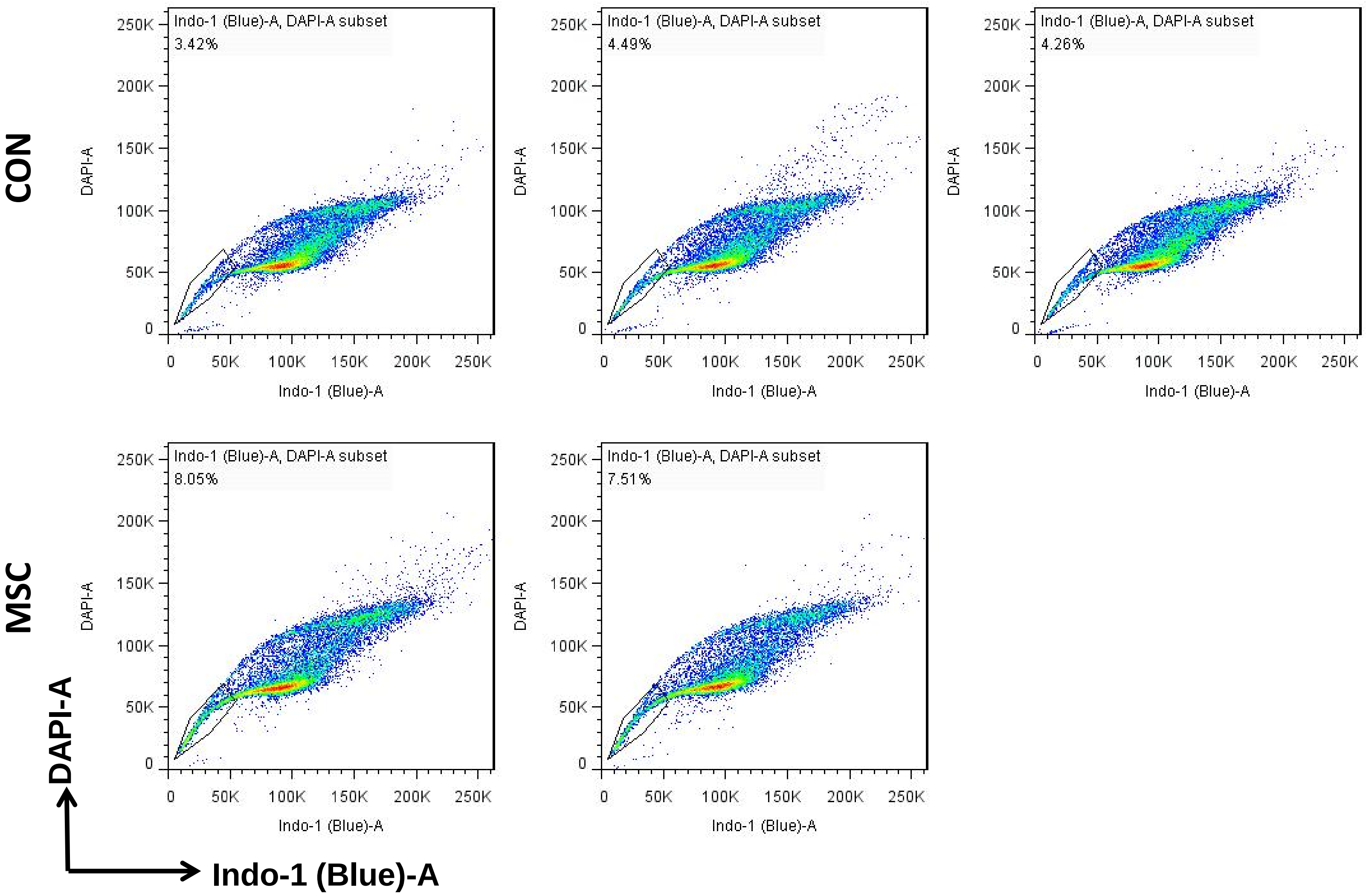

Supplementary Data 4
